# The cost-effectiveness of systematic screening for age-related macular degeneration in South Korea

**DOI:** 10.1371/journal.pone.0206690

**Published:** 2018-10-31

**Authors:** Ra Ho, Lina D. Song, Jin A. Choi, Donghyun Jee

**Affiliations:** 1 Department of Ophthalmology and Visual Science, Bucheon St. Mary’s Hospital, College of Medicine, The Catholic University of Korea, Bucheon, South Korea; 2 PhD Program in Health Policy, Harvard University, Cambridge, Massachusetts, United States of America; 3 Department of Ophthalmology and Visual Science, St Vincent’s Hospital, College of Medicine, The Catholic University of Korea, Seoul, South Korea; Singapore National Eye Centre, SINGAPORE

## Abstract

**Background:**

Interventions that can facilitate early diagnosis of age-related macular degeneration (AMD) will facilitate early treatment and improve clinical outcomes but there has been concerns about additional medical costs to the health care system. An examination through a retina fundus photography by a non-specialist has been suggested as a potential cost-effective alternative to a direct examination by a specialist, but limited scientific data exists on the cost-effectiveness of screening strategies for AMD. Our objective is to conduct an economic evaluation of various population-wide screening strategies for AMD among the South Korean population.

**Methods and findings:**

Using a Markov cohort model, we evaluated the cost-effectiveness of four AMD screening strategies (opportunistic examination, opportunistic treatment, systematic photography, and systematic examination) in comparison with status quo (no screening) for South Korean adults. We projected a life time horizon to study a hypothetical cohort of 100,00 persons of age 40 with and without AMD at baseline. The outcome measures were quality-adjusted life-years (QALYs) gained, cost from the societal perspective, and the incremental cost-effectiveness ratio (ICER) of each strategy. Interventions were evaluated at a willingness-to-pay (WTP) threshold of 30,000,000 KRW ($27,538) per QALY gained. Deterministic and probabilistic sensitivity analyses were conducted to address the model uncertainty. Opportunistic examination was strongly dominated because it generated fewer expected QALYs but incurred greater expected cost than the other screening strategies. The mean lifetime expected costs were 289,013 KRW, 363,692 KRW, 9,351,964 KRW, and 12,309,783 KRW, and the mean QALYs gained were 37.73, 37.75, 40.47, 40.68, for no screening, opportunistic treatment, systematic photography, and systematic examination, respectively. The results were most sensitive to the utility weight of mild AMD, the probability of complication from treatment, the cost of being in mild AMD, and the probability of recovery from complication. After eliminating the two weakly dominated strategies, systematic photography was cost-effective at the ICER of 3,310,448 KRW per QALY in comparison to status quo.

**Conclusions:**

Under the WTP threshold of 30,000,000 KRW per QALY, systematic photography is cost-effective for screening AMD in South Korean adults. Systematic examination by ophthalmologists generates more expected QALY and cost compared to systematic photography.

## Introduction

Age-related macular degeneration (AMD), a deterioration or loss of the central portion of the retina, is the leading cause of visual impairment and blindness worldwide [1–3]. The global prevalence of early, late, and any AMD is estimated to be 8.01%, 0.37%, and 8.69%, respectively, and the number of people with AMD is projected to reach 288 million by 2040 [4]. The societal burden of AMD is significant. Not only does it decrease the patients’ quality of life, it also increases the health care cost [5–8]. In the United States alone, the direct medical costs of AMD among adults aged 40 years or older is estimated to be $575 million [9].

Most AMD cases are diagnosed when a patient seeks care after experiencing ocular symptoms, at which point there is a greater chance that he or she has already progressed to severe states [10–12]. Interventions that can facilitate early diagnosis will facilitate early treatment which can delay symptoms or make them less severe [5, 6, 13–18], but there is a concern that the cost of screening will add direct medical costs. Recently, examinations through a retina fundus photography by a non-specialist has been suggested as a potential cost-effective alternative to a direct examination by a specialist. However, there has been debates about the cost-effectiveness, and limited data exists [14, 19]. For example, in the United Kingdom, the NHS diabetic eye screening program offers digital fundus photograph for all 12 years old or more patients with diabetes [20]. Moreover, recent study reported that the addition of optical coherence tomography to retinal screening program in UK can result in more cost effective [21].

South Korea has recently been experiencing a substantial and increasing disease burden of AMD. A nationwide cross-sectional study shows that the prevalence of AMD in the South Korean population is 6.6% [22], higher than other Asian countries and comparable to the ones of Western countries [4, 23]. With the aging of the South Korean population, the AMD incidence is expected to rapidly grow. The National Health Insurance (NHI) program of South Korea has been providing regular health examinations without cost-sharing to all beneficiaries starting at age 40 [24]. Although these visits can be an opportunity for population-wide screening for common ocular diseases such as glaucoma and AMD, ocular tests are currently not included in the screening program.

Therefore, we hypothesized that the addition of digital retinal fundus photography to regular health examination by NHI would be cost-effective screening method. Our objective is to conduct an economic evaluation of various population-wide screening strategies for AMD among the South Korean elderly population. We constructed a Markov model to project the health benefits and costs of four screening strategies (opportunistic examination, opportunistic treatment, systematic photography, and systematic examination) in comparison with status quo (no screening) for age-related macular degeneration (AMD) among the South Korean male and female patients aged 40 years with and without AMD.

## Methods

### Overview

We developed a Markov model based on previous models to assess the health benefits and health care costs associated with each intervention. Taking a societal perspective, we compared the incremental cost-effectiveness of each interventions with one another to calculate the cost-effectiveness. All clinical, utility, and economic parameters are based either on published sources or expert opinions. The parameter values and the ranges for sensitivity analyses are listed in Table 1. TreeAge Pro 2016 Health Care (TreeAge Software) was used for analysis.

**Table 1:** Model parameters.

	Name	Description	Value	Reference
Demographic	pNotExamined	Initial proportion of people not being examined opportunistically	0.34	[22]
	Prevalence	AMD prevalence	0.066	[22]
	pNoToMild	AMD incidence rate	0.01	[25]
	age	Initial cohort age	40	assumption
Disease progression	pMildToMod	Probability of transition from mild AMD to miderate AMD	0.0022	[26]
	pModToSev	Probability of transition from moderate AMD to severe AMD	0.0015	[26]
	pImprove	Probability of regressing to less severe state	0.1	[27]
	effectTx	Probability of slowing down the disease progression under treatment	0.5	[28]
	pTxComp	Probability of developing complication given treatment	0.005	[29, 30]
	pRecov	Probability of recover after complication given treatment	0.4	[29, 30]
Others	Spec	Specificity of photography	0.79	[31]
	Sens	Sensitivity of photography	0.86	[31]
	WTP	Willingness to pay threshold	30,000,000	assumption
	r	discount rate	0.05	assumption
Utility	U_noAMD	Utility of no AMD	1	[28, 32]
	U_mildAMD	Utility of mild AMD	0.81	[28, 32]
	U_modAMD	Utility of moderate AMD	0.57	[28, 32]
	U_sevAMD	Utility of severe AMD	0.51	[28, 32]
	U_Cx	Utility of complication	0.4	[28, 32]
Cost	C_noAMD	Cost of no AMD	0	calculation
	C_mildAMD	Cost of mild AMD	620,702	calculation
	C_modAMD	Cost of moderate AMD	1,757,690	calculation
	C_sevAMD	Cost of severe AMD	5,145,366	calculation
	C_low_vision	Cost of low vision	1,000,000	assumption
	C_additonal_Cx	Additional cost from treating complication	3,000,000	assumption
	C_fundusphoto	Cost of fundus photography	8930	calculation
	C_examination	Cost of systematic examination	111,958	calculation

### Study population

Our study population is a 100,000 hypothetical cohort of South Korean male and female patients aged 40 years with and without AMD. We chose age 40 as the starting age because this population account for the majority of the disease and the South Korean National Health Insurance (NHI) starts providing free health examinations at age 40, which makes it ideal to initiate the ocular examinations. We projected the model until the patient either dies or reaches age 100 years to estimate the AMD-related health care cost throughout the lifetime.

### Markov model

Our Markov model has 5 relevant states for AMD progression: no AMD, mild AMD, moderate AMD, severe AMD, and death. The AMD severity was categorized into three levels according to visual acuity [25, 28, 33]. At each cycle, a patient accumulates health utility and cost based on the disease state he or she is in, and the patient either stays in the same state or transitions to a different state according to the transition probabilities associated with the current health state. The cycle length was 1 year.

The initial population is distributed across the health states according to the AMD prevalence of the South Korean population [22]. We assumed that untreated patients can only progress from less severe to more severe disease states, but the treated patients can regress from more severe to less severe disease states. We assumed that the mortality depended only on the patient’s age and there was no additional disease specific risk from AMD. The age-specific mortality rate was obtained from 2015 Korean life table.

### Overview of screening strategies

#### No screening

A patient receives neither a screening examination nor any treatment, and the AMD progresses according to the natural disease progression rate.

#### Opportunistic examination

When a patient presents himself to an ophthalmologist for a general ocular examination or any symptoms, an ophthalmologist performs an examination to screen for AMD. If AMD is diagnosed, the patient is treated and managed annually according to the guideline. The opportunistic examination strategy only examines the population who are initially linked to the providers, instead of actively screening the entire population, and thus the remaining population are left unscreened. The proportion of patients who present themselves to an ophthalmologist for any reasons was estimated from observational studies [22]. The decision to visit an ophthalmologist recurs every year, and the patient goes through the recommended treatment once AMD is diagnosed.

#### Opportunistic treatment

When a patient presents himself to an ophthalmologist for AMD related symptoms, he goes through an examination to screen for AMD. If AMD is diagnosed, the patient is treated and managed annually according to the guideline. The difference between opportunistic examination and opportunistic treatment is that only patients who are actively experiencing the AMD related symptoms such as change in the quality of vision qualify for an examination.

#### Systematic photography

The entire study population is annually screened for AMD via fundus photography starting at age 40. The screening test is imperfect, and there are false positives as well as true negatives. Test negative patients undergo no further screening or treatment, whereas the test positive patients are further examined to confirm diagnosis, and go under treatment and management if confirmed positive.

#### Systematic examination

All population undergo annual examinations for AMD by ophthalmologists starting at age 40. The diagnosis from an ophthalmologist is assumed as the gold standard. AMD diagnosed patients undergo treatment and management according to the guidelines.

### Model assumptions

#### Disease prevalence and progression rate

The AMD prevalence for the initial distribution was obtained from a published epidemiologic study for South Korean population [22]. We obtained the disease transition probabilities from randomized trials and observational studies [26, 34, 35]. To account for the potential differences in the study population, we conducted extensive sensitivity analyses on these parameters.

#### Treatment effectiveness

The mechanism through which screening can improve the health outcome is that early screening and detection allow early treatment of AMD, which both slows down the disease progression and improves the conditions for some patients. Evidence shows that effective treatment such as anti-vascular endothelial growth factor therapy can stabilize or delay the deterioration in vision in most cases of AMD [36–38]. Thus, under treatment strategy, the disease transition probability is reduced and there is a chance of regressing to no AMD state, which was zero for the untreated cohort. The treatment effectiveness and disease regression parameters were estimated from published sources were varied in the sensitivity analyses [28, 34, 35].

#### Complication

Our model incorporates the possibility that patients who receive treatment may develop complications or side-effects from treatment [34]. We assumed that all patients who are under treatment are equally likely to develop complications. Once they develop complications, the patient accumulates additional cost of treatment and the quality of life is reduced [39]. There is no increased chance of death, and once the patient recovers, he goes back to his original disease state.

#### Test characteristics

The test characteristics (sensitivity and specificity) for fundus photography were obtained from published literature [14]. An examination by an ophthalmologist was considered the gold-standard, thus having perfect sensitivity and specificity.

#### Costs

We took a societal perspective to examine the impact of providing nation-wide AMD screening through the Korean National Health Insurance. We included both direct and indirect health care costs related to AMD treatment and management. The direct costs of AMD diagnosis and treatment were based on the reimbursement rates for related procedures from the 2016 average Korean National Health Insurance Data. Direct medical costs of care included the costs of inpatient and outpatient visits to ophthalmologists, tests to monitor patients, and the screening costs. We also included the indirect costs of patients’ and providers’ time associated with the visits and the transportation costs. We did not include the indirect societal cost from the loss of productivity due to impaired vision, as these are incorporated into the QALYs. S1 Table shows a detailed breakdown of the cost estimation. All costs were adjusted to 2018 South Korean Won (KRW) using the Consumer Price Index.

#### Utilities

The health benefit was measured as quality-adjusted life-years (QALYs), the value of a year of life taking into account the disutility from experiencing AMD. The total accrued QALYs were calculated by summing the total expected life years, where each year is scaled by the utility weights based on the disease state. The utility weights for living with AMD were based on published studies linking visual acuity and patient utilities [40], and all utility weights were varied in sensitivity analyses.

### Model validation

The internal validity of the model was assessed by examining the natural history of the unscreened patients (S1 Fig). The external validity of the model was assessed by comparing the disease progression of AMD patients projected from the model to the observational data that were not used to inform the model parameter.

### Economic analysis

Using the projected QALYs and costs, we calculated an incremental cost-effectiveness ratio (ICER), the incremental change in the AMD associated costs divided by the incremental QALYs, for each screening strategy with respect to status quo. Future costs and QALYs were discounted to the present value at 5% annual discounting rate. The ICER was compared to a cost-effectiveness threshold of 30,000,000 KRW/QALY, an approximate GPD per capital of South Korean in 2017.

### Sensitivity analysis

We conducted multiple sensitivity analyses to examine how the parameter uncertainty affects our results. First, in a univariate analysis, each individual model parameter was varied over the parameter range, and the net monetary benefit of the dominant strategy was calculated at the willingness-to-pay threshold of 30,000,000 KRW/QALY. Next, incorporating the results from the univariate analysis and also consulting with clinical experts, two-way sensitivity analysis was performed. Finally, to address the stochastic uncertainty in parameters, we conducted a probabilistic sensitivity analysis by pulling 10,000 random draws from a probability distribution on select key parameters, where the parameter distributions were estimated from published studies and expert opinion [34, 35, 41].

## Results

### Model validation

We validated our model results by comparing the natural history of AMD progression to that of an observational study not used to inform the model parameters [26]. In an observational study of U.S. cohort with and without AMD,the combined incidence of early or late AMD over the 20-year period was 28.9%. This is consistent with the disease progression of our model cohort where any-stage AMD prevalence increases from 7% to about 30% in the first 20 years under the "opportunistic examination" strategy, which most likely resembles the care utilization patterns the population in the Beaver Dam Eye Study [26]. The treatment effectiveness was consistent with the results of the randomized trial, where there are about 30% fewer patients who have progressed from wet AMD during the first 5 years of diagnosis [34, 41, 42].

### Base model

**Fig 1:**
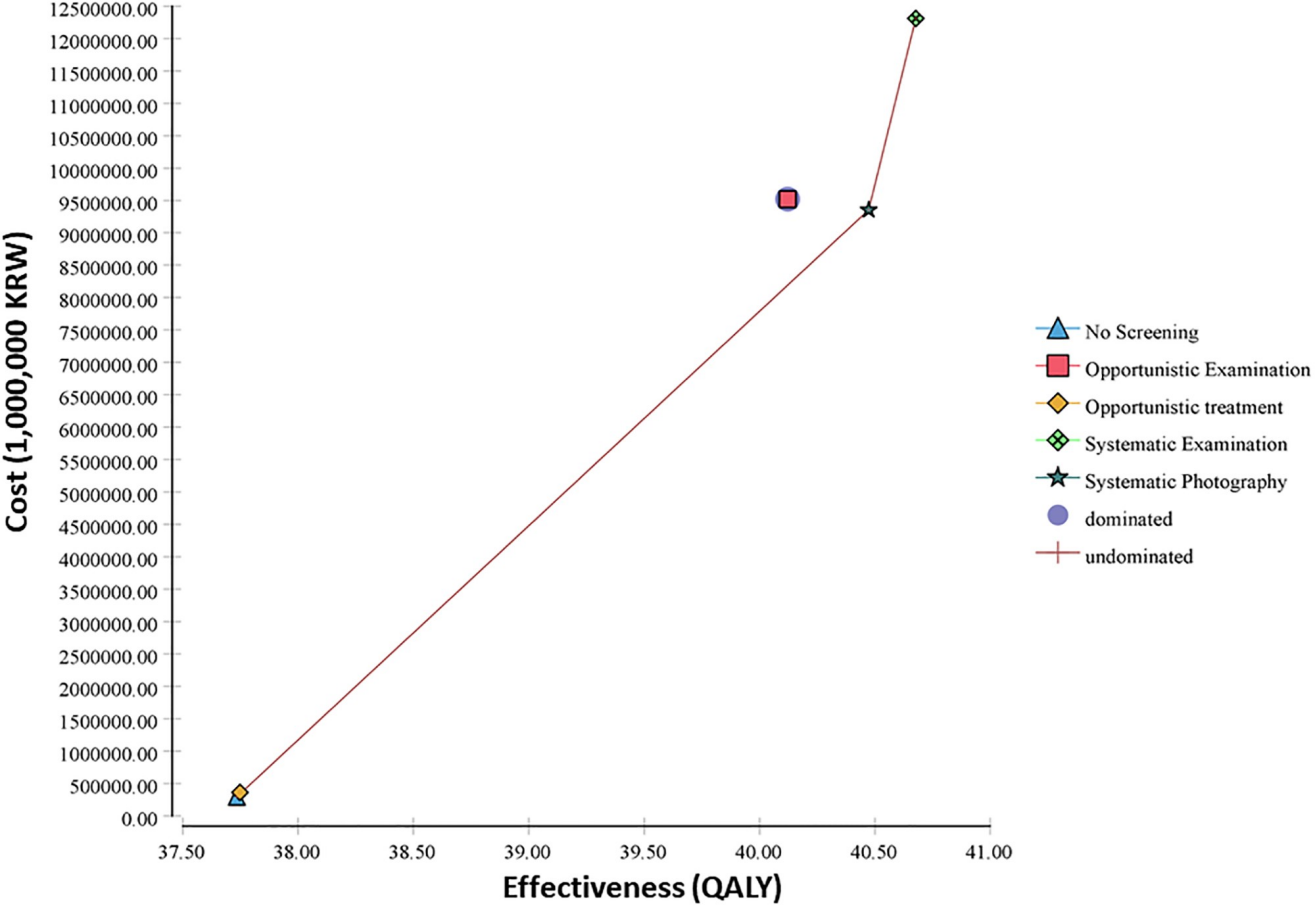
Cost-effectiveness plane comparing the effectiveness of status quo (no screening) and the four screening strategies (opportunistic examination, systematic examination, systematic photography, and opportunistic treatment).

Fig 1 and Table 2 report the main result, cost-effectiveness of status quo and four screening strategies for AMD. Under status quo (no screening), the total expected medical cost associated with AMD is 289,013 KRW and the total expected QALYs gained is 37.73 for a 40-year old patient throughout the lifetime. Among the four screening strategies, opportunistic examination is strongly dominated by other strategies because it generates fewer QALYs (40.12 QALYs) yet incurs greater costs (9,520,867 KRW) than the other strategies. Opportunistic treatment and systematic examination are weakly dominated by systematic photography. After ruling out all dominated strategies, systematic photography incurs expected costs of 9,351,964 KRW and QALYs of 40.47, yielding an ICER of 3,310,448 KRW per QALY gained. Considering our willingness-to-pay threshold of 30,000,000 KRW, systematic examination is extremely cost-effective.

**Table 2:** Cost-effectiveness of intervention strategies for AMD screening (A) all strategies (top) and (B) excluding dominated strategies (bottom).

**A**					
**Strategy**	**Cost**	**Incr Cost**	**Eff**	**Incr Eff**	**Incr C/E**
No screening	289,013.77		37.73		0
Opportunistic treatment	363,692.85	74,679.08	37.75	0.01	5,688,399.32
Systematic photography	9,351,964.25	8,988,271.41	40.47	2.72	3,298,990.77
Opportunistic examination	9,520,867.26	168,903.00	40.12	-0.35	-480,783.88
Systemic examination	12,309,783.34	2,957,819.08	40.68	0.20	14,505,241.49
**B**					
**Strategy**	**Cost**	**Incr Cost**	**Eff**	**Incr Eff**	**Incr C/E**
No screening	289,013.77		37.73		
Systematic photography	9,351,964.25	9,062,950.49	40.47	2.74	3,310,448.97
Systematic examination	12,309,783.34	2,957,819.08	40.68	0.20	14,505,241.49

### Sensitivity analysis

**Fig 2:**
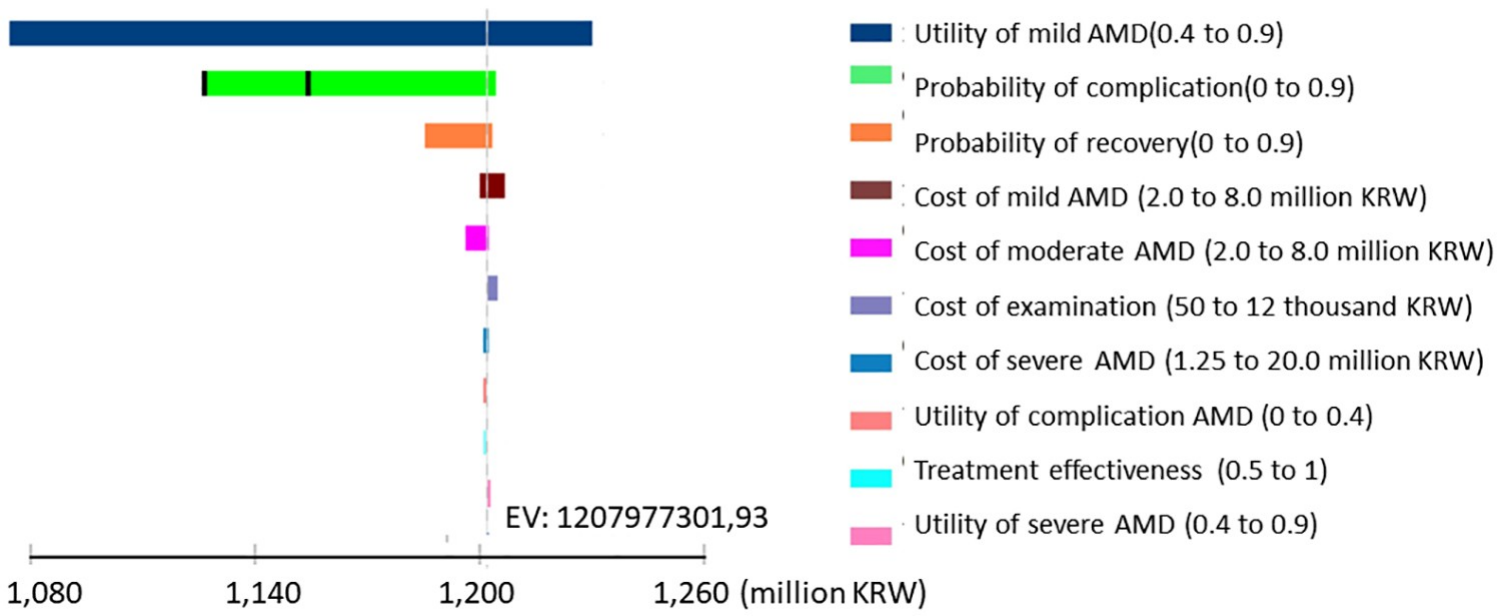
Tornado diagram of the range of net monetary benefits at willingness-to-pay threshold 30,000,000 KRW per QALY for all major model parameters.

We first conducted multiple univariate sensitivity analyses for each parameter at willingness-to-pay threshold of 30,000,000 KRW, represented as a tornado diagram (Fig 2). The width of the bars in the tornado diagram represents the impact of each parameter on the model result, where the outcome is the net monetary benefits when strategies were compared at WTP threshold 30,000,000 KRW. The results are presented in a decreasing order from the top. Among the model parameters, results were most sensitive to the utility weight of mild AMD, the probability of complication from treatment, the cost of being in mild AMD, and the probability of recovery from complication. We conducted an additional one-way sensitivity analysis on the most influential parameters identified from the tornado diagram. In general, our results were most sensitive to the complication rate and the recovery rate. None of the parameters examined in the univariate analyses produced the result qualitatively different from our main conclusion.

**Fig 3:**
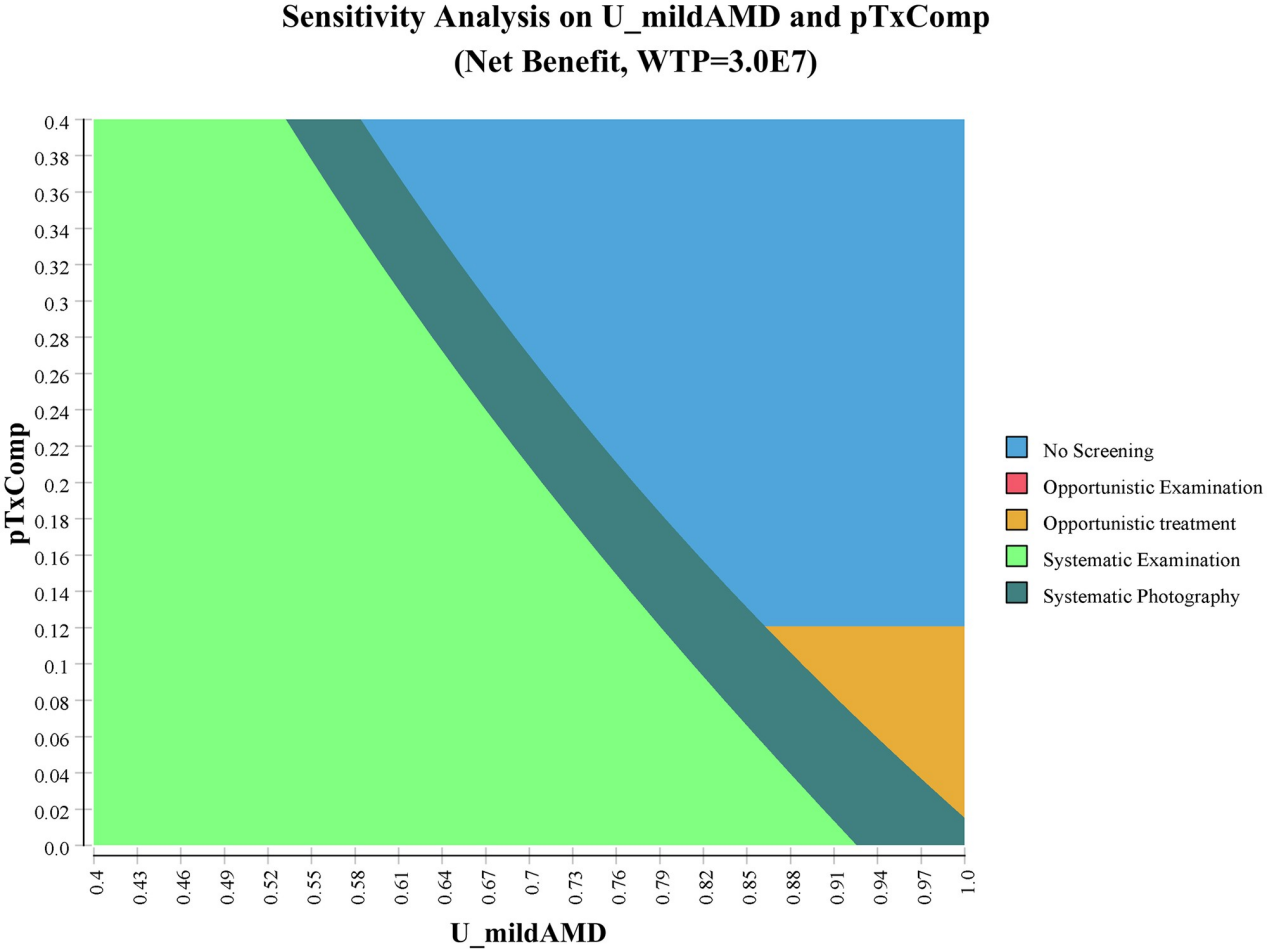
Two-way sensitivity analysis simultaneously varying the utility weight of mild age-related macular degeneration and the complication rate of treatment.

Because the assumptions on the utility weight of mild AMD, the probability of complication from treatment, the cost of being in mild AMD, and the probability of recovery from complication are critical in the way our model reflects the differences among interventions, we conducted two-way sensitivity analyses on these parameters. Fig 3 and S2–S4 Figs show that when the complication rate of treatment is high and the utility weight of mild AMD is high, no screening becomes the dominant strategy. When the complication rate of treatment and the utility weight of mild AMD are both low, systemic examination becomes dominant. However, it is unlikely that the complication rate of treatment of anti-vascular endothelial growth factor therapy becomes greater than 5% and the utility weight of mild AMD becomes lower than 0.6, and therefore systematic photography is a more cost-effective strategy under the reasonable range of parameter values.

**Fig 4:**
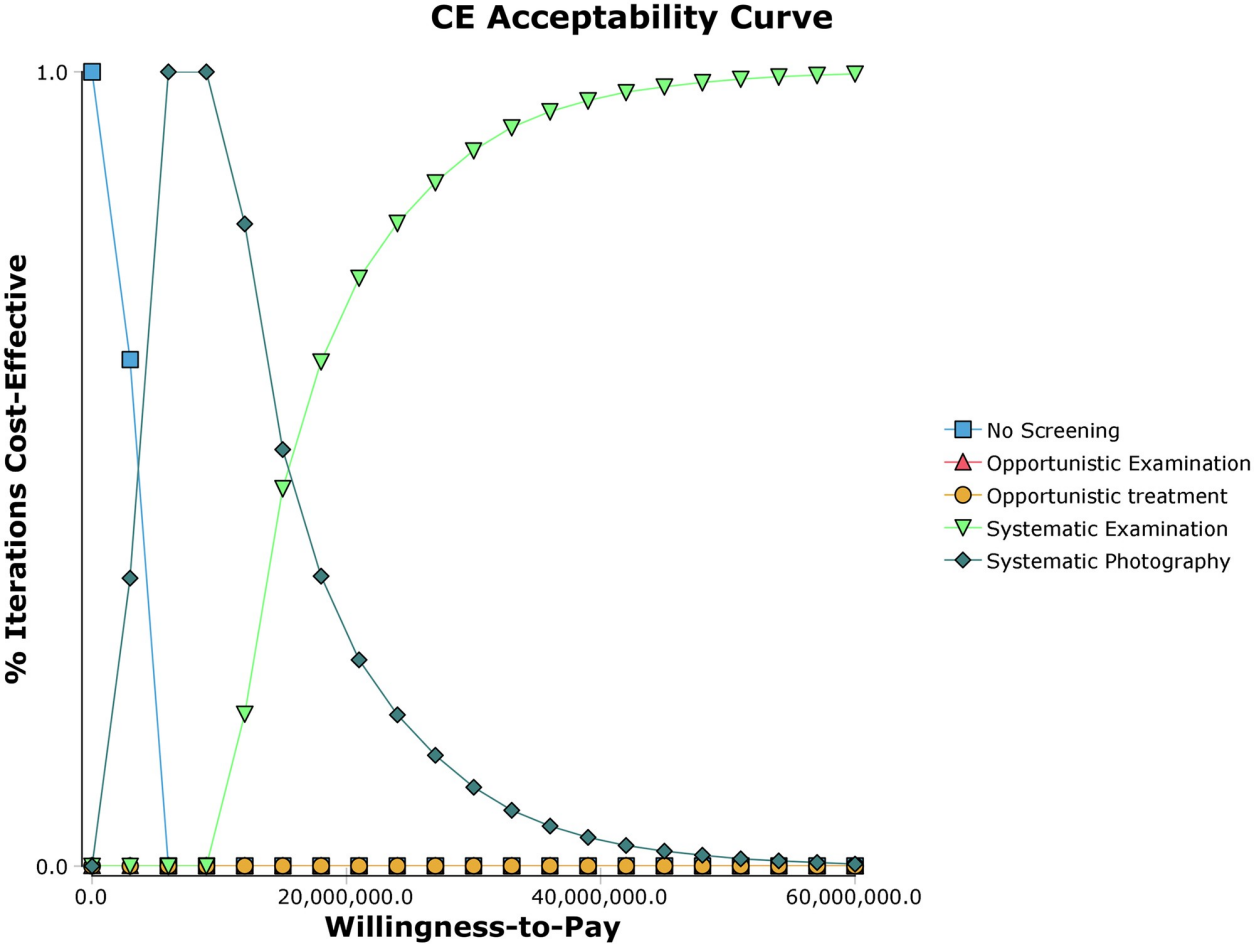
Cost-effectiveness acceptability curve from the probabilistic sensitivity analysis.

We also conducted a probabilistic sensitivity analysis accounting for the parameter uncertainty, where the probabilities of each strategy being cost-effective was plotted against the WTP threshold (Fig 4). The probability that status quo becomes the dominant strategy increases when the WTP threshold is below 5,000,000 KRW/QALY. The probability that systemic photography becomes the dominant strategy increases when the WTP threshold is between 5,000,000 and 15,000,000 KRW/QALY. However, under the current WTP threshold of 30,000,000 KRW/QALY, the probability of systematic examination being the dominant strategy gets close to 1.

## Discussions

Although retinal screening is an effective method for early detection and prevention of AMD, there has been limited scientific data on the cost-effectiveness of various screening strategies. Our study finds that screening through systematic photography for the entire South Korean population over 40 years old is a highly cost-effective screening strategy with an ICER of 3,310,448 KRW per QALY. Other strategies such as opportunistic examination, systematic photography, and opportunistic treatment, are weakly or strongly dominated by the systematic examination strategy. Based on the World Health Organization (WHO)’s cost-effectiveness threshold of 1 to 3 times the GDP per capita of the country, systematic photography is highly cost-effective among Korean population. The ICER varied based on some key parameter values, such as the utility weight of mild AMD, the probability of complication from treatment, the cost of being in mild AMD, and the probability of recovery from complication. When the complication rate of treatment and the utility weight of mild AMD were high, no screening became the dominant strategy. In general, however, the results of sensitivity analyses show that under reasonable ranges of the parameter values, systematic photography was the dominant strategy for our study population. There has been an ongoing debate about the cost-effectiveness of systematic photography vs. systematic examination as a primary screening modality for AMD [14, 19]. One study reported that screening using fundus photographs by ophthalmologist showed highly effectiveness [43]. Another study reported that the addition of optical coherence tomography can save cost without reducing health benefits, which improve the cost-effectiveness of retinal screening programs in the UK [21]. Systematic photography can be potentially cost-effective since it can save the direct examination cost by the ophthalmologist. For example, extending public health coverage to eye examination by optometrists is cost-effective based on a commonly used threshold of $50,000/QALY [44]. Our study shows that the addition of digital retinal fundus photography to regular health examination by NHI is highly cost-effective screening method.

There are several key assumptions to note. First, we assumed that under status quo, patients do not receive any screening or treatment throughout the lifetime, but it is unlikely that the patients remain untreated once they experience significant vision loss. As an alternative scenario, we examined the opportunistic treatment strategy which assumed that patients who have symptomatic AMD will get diagnosed and treated. The current care seeking pattern of the Korean population will probably be a combination of no screening and opportunistic treatment. Our result shows that in comparison to opportunistic treatment, all three other screening strategies are cost-effective, and systematic photography is the most cost-effective option. We also assumed perfect adherence to follow-up. However, in reality, those without AMD or those who recovered from AMD may not adhere to annual follow up. In particular, there has been a concern for the suboptimal adherence to screenings among the South Korean elderly population in general, even if there is no cost-sharing for patients [45]. Since our population benefit most from screening and initial diagnosis rather than the follow up process, this will mitigate our concern. Finally, we assumed the examination by an ophthalmologist as a gold-standard, but misdiagnosis can still occur in real practice settings, which may reduce the cost-effectiveness of systematic examination.

There are other limitations from the assumptions we made on the model structure and the parameters. We did not consider the interactive effects between the parameters. Each of the model parameter value we used reflects the population average, ant it is possible that some of these parameters are not independent of each other. We believe the impact of the interactive effects between some model parameters on our results would be minimal, as the patients most likely to experience these effects (e.g., those simultaneously in advanced age and AMD stage) are only a small fraction of the study population. We also did not specify gender differences or age-specific risk of AMD. Such assumptions will make the estimated outcomes less variable than if parameters depended on age or gender, but the average effect will not be significantly affected. We also note that the underlying population from which some of our model parameters were estimated may differ clinically from our study population. The probability of visiting an ophthalmologist for opportunistic screening was assumed constant across disease state, but the patients in severe AMD may be more likely to visit an ophthalmologist. These assumptions were imposed to keep our model relatively simple and only reflect the key differences among the intervention strategies, thereby reducing the potential biases from the modeler. Lastly, the model and conclusion of the present study are specific to the values used in the assumption. These values likely differ between different system or countries, and would change if the cost of treatment dramatically changed. Similarly different results and conclusions may be derived if these values were modified for another setting.

Currently, the universal health care system of South Korea provide screening for services such as a medical interview and postural examination, chest X-ray examination, blood test, urine test, and dental screening for free. However, screening for ocular conditions is not included as a part of general health examination. Our study shows that covering screening for AMD through the population level systematic photography is cost-effective at reducing the future AMD burden. There are also possibilities of co-examining from systemic photography for other common ocular disease such as glaucoma or diabetic retinopathy that also incurs high burden to the South Korean elderly population, which will make the systematic photography even more cost-effective. A recent study demonstrated that a simultaneous retinal screening for both AMD and diabetic retinopathy would be cost effective in a Hong Kong public health care setting [46].

## Conclusion

Despite the above mentioned limitations, our study shows that covering eye examination by fundus photography as part of screening services will increase expected QALYs to the South Korean elderly population at low ICER. The prevalence and incidence of AMD among South Koreans is higher than other Asian countries, and responding to the high economic burden of AMD epidemic is imperative. Our result shows that providing AMD screening through systematic photography can potentially mitigate the future disease burden and cost of AMD among South Korean population. Findings from the study should be tested in prospective studies to see if the findings are validated and then may be incorporated into policy changes.

## Supporting information

S1 Fig Markov tracings of age-related macular degeneration severity over time by intervention type. (TIF)

S2 Fig Two-way sensitivity analysis simultaneously varying the complication rate and the recovery rate following treatment. (TIF)

S3 Fig Two-way sensitivity analysis simultaneously varying the utility weight and the cost of mild age-related macular degeneration. (TIF)

S4 Fig Two-way sensitivity analysis simultaneously varying the cost of mild and moderate age-related macular degeneration. (TIF)

S1 Table Detailed breakdown of costs. (DOCX)
