# Correction: The cost-effectiveness of systematic screening for age-related macular degeneration in South Korea

**DOI:** 10.1371/journal.pone.0207736

**Published:** 2018-11-15

**Authors:** Ho Ra, Lina D. Song, Jin A. Choi, Donghyun Jee

The first author’s name is spelled incorrectly. The correct name is: Ho Ra. The correct citation is: Ra H, Song LD, Choi JA, Jee D (2018) The cost-effectiveness of systematic screening for age-related macular degeneration in South Korea. PLoS ONE 13(10): e0206690. https://doi.org/10.1371/journal.pone.0206690
