# Correction: The cost-effectiveness of systematic screening for age-related macular degeneration in South Korea

**DOI:** 10.1371/journal.pone.0209521

**Published:** 2018-12-14

**Authors:** Ho Ra, Lina D. Song, Jin A. Choi, Donghyun Jee

In the Funding section, the second grant number from the funder Ministry of Health & Welfare, Republic of Korea is listed incorrectly. The correct grant number is: HC17C0133.
