# Supplementary figures and images for: The cost-effectiveness of systematic screening for age-related macular degeneration in South Korea
Source: PLoS One. 2018 Oct 31;13(10):e0206690. doi: 10.1371/journal.pone.0206690 (PMC6209376; doi:10.1371/journal.pone.0206690)

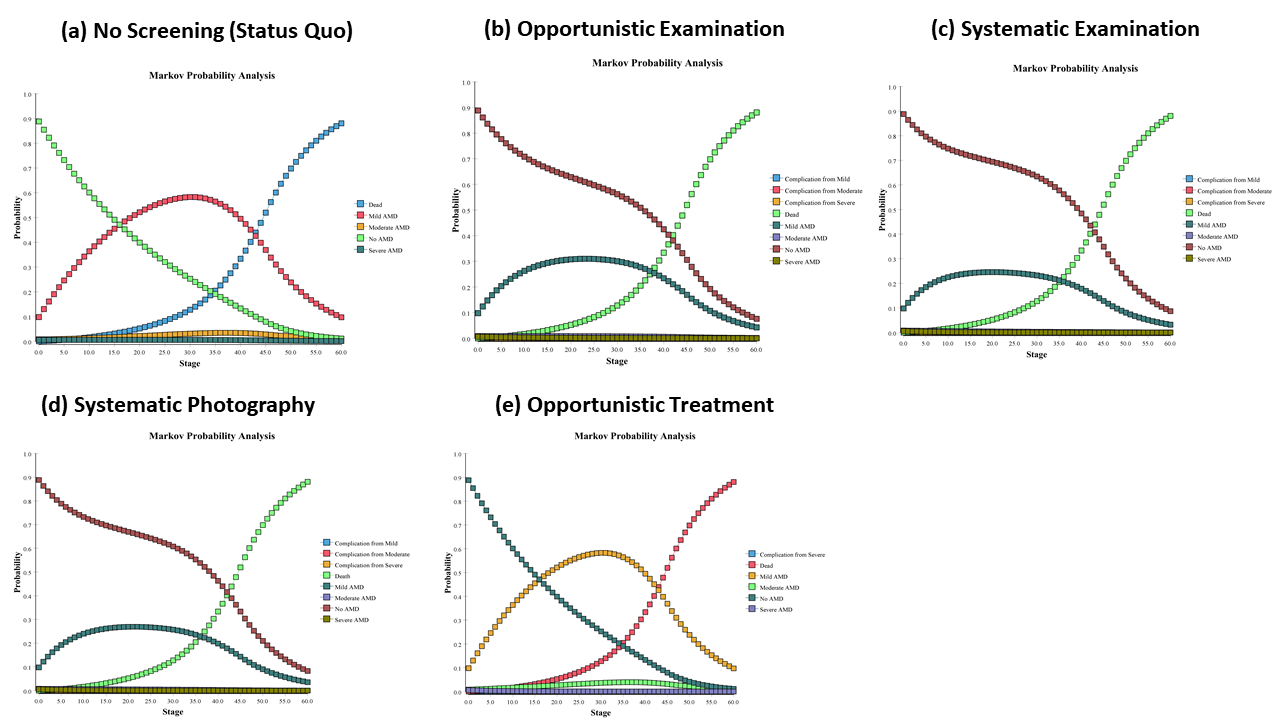

Supplement: S1 Fig — (TIF) [file pone.0206690.s001.tif]

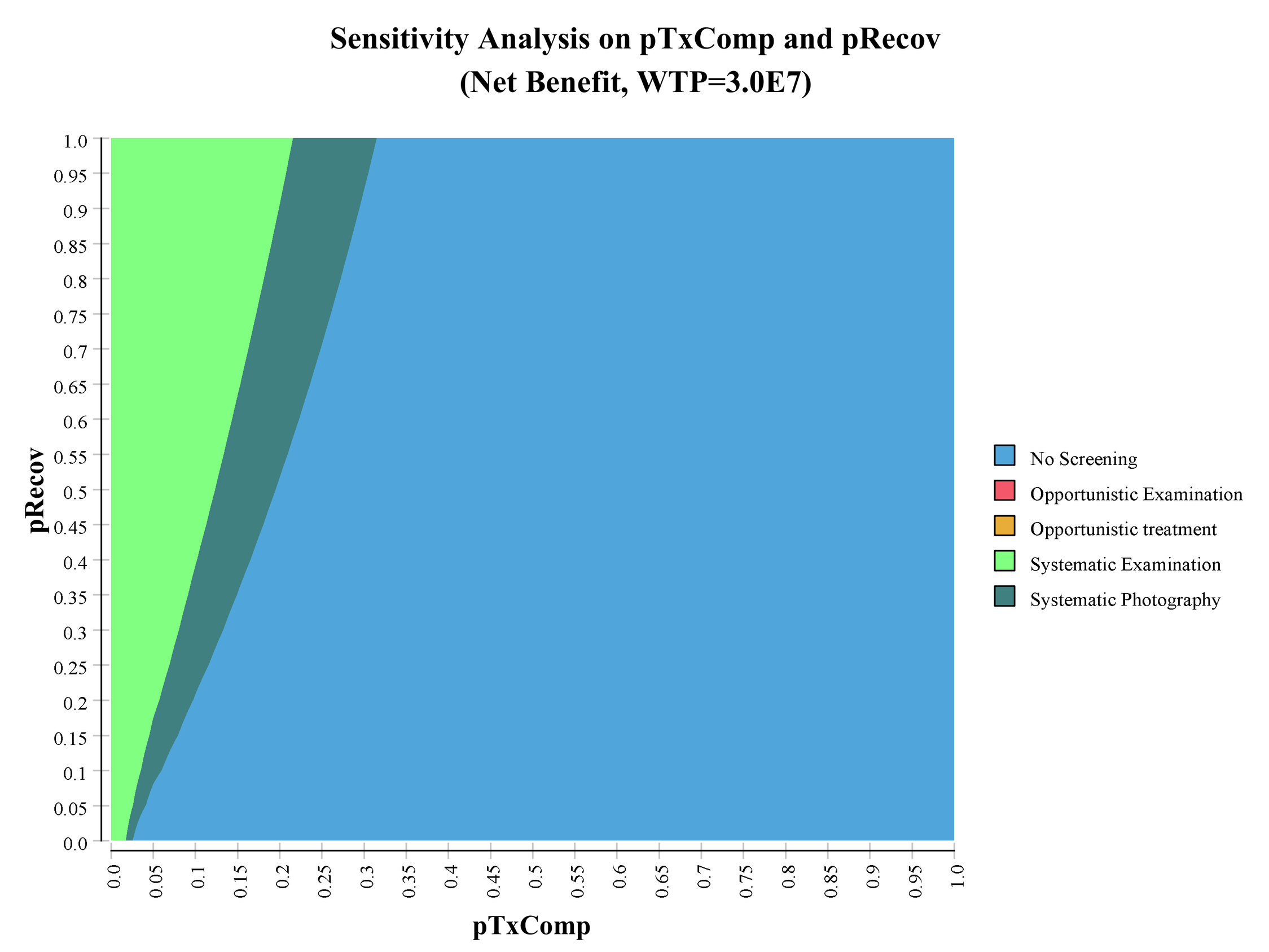

Supplement: S2 Fig — (TIF) [file pone.0206690.s002.tif]

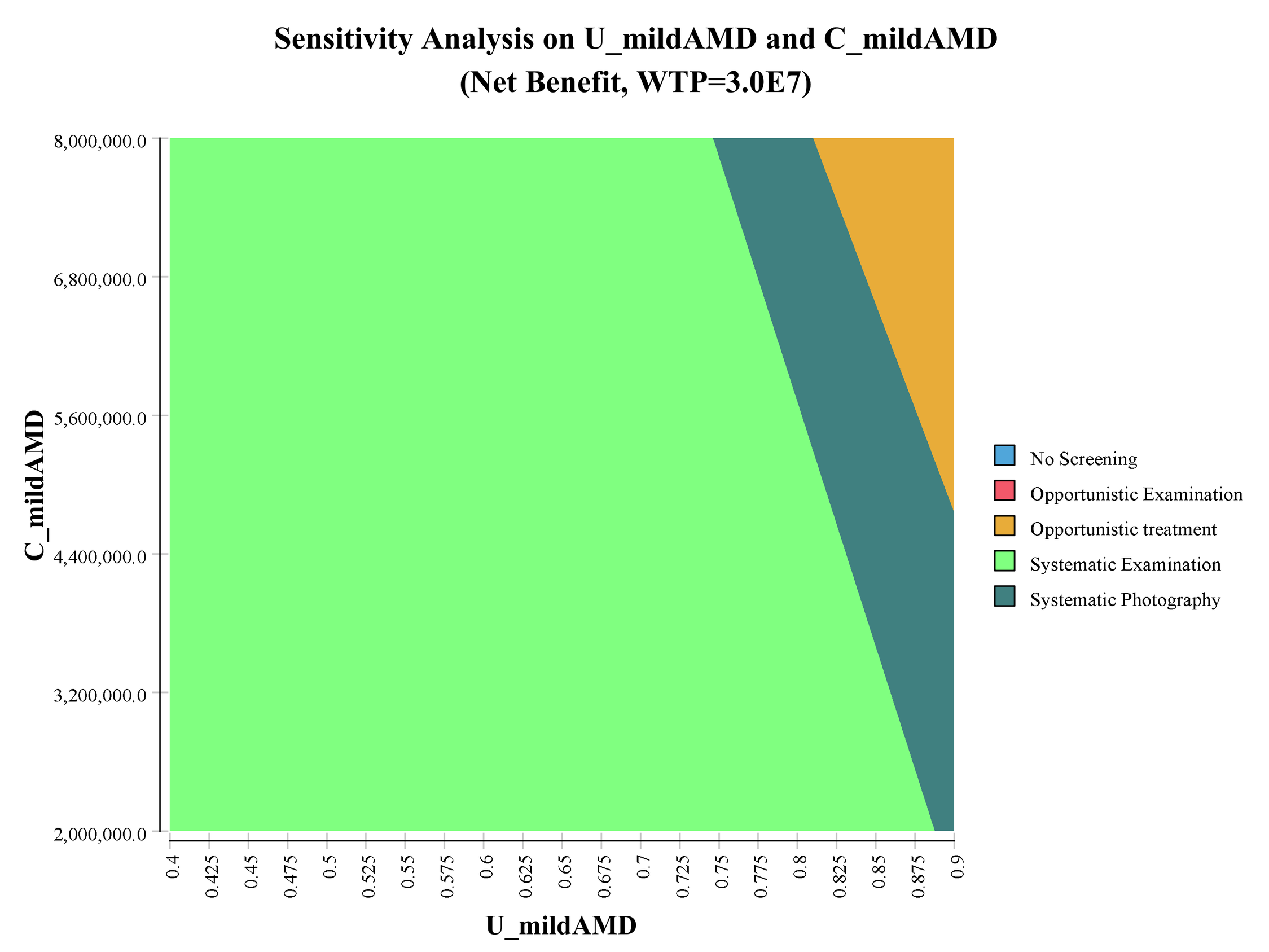

Supplement: S3 Fig — (TIF) [file pone.0206690.s003.tif]

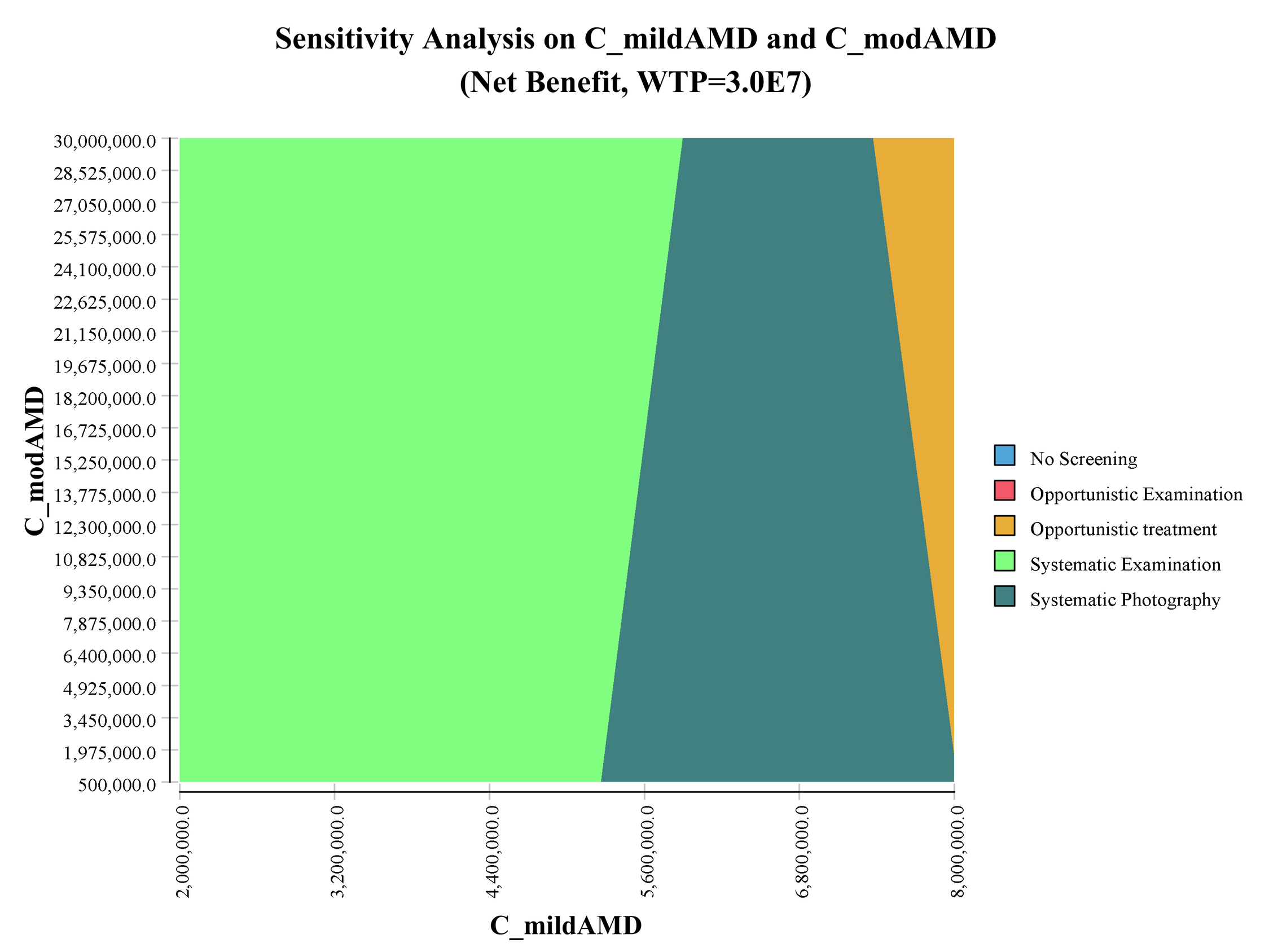

Supplement: S4 Fig — (TIF) [file pone.0206690.s004.tif]
