# Supplementary material for: The cost-effectiveness of systematic screening for age-related macular degeneration in South Korea
Source: PLoS One. 2018 Oct 31;13(10):e0206690. doi: 10.1371/journal.pone.0206690 (PMC6209376; doi:10.1371/journal.pone.0206690)
Supplement: S1 Table — (DOCX) [file pone.0206690.s005.docx]

**Table S1. Detailed breakdown of costs**

|  |  | **Total Annual Cost (KRW)** |
| --- | --- | --- |
| **Medical cost** | Direct medical cost | 4145366 |
|  | Indirect medical cost | 172871 |
| **Non-medical cost** | Time cost | 99631 |
|  | Transportation cost | 15848 |
|  | Nursing cost | 28401 |
| **Cost of screening** | Medical cost | 8930 |
|  | Time cost | 8930 |
| **Cost of provider** | Direct medical cost | 111958 |
|  | Time cost | 24908 |
|  | Transportation cost | 3962 |
|  | Nursing cost | 7100 |
